# Defect Detection in Printed Circuit Boards Using Semi-Supervised Learning

**DOI:** 10.3390/s23063246

**Published:** 2023-03-19

**Authors:** Thi Tram Anh Pham, Do Kieu Trang Thoi, Hyohoon Choi, Suhyun Park

**Affiliations:** 1 Department of Electronic and Electrical Engineering, Ewha Womans University, Seoul 03760, Republic of Korea; 2 Pixel Inc., Pyeongtaek 17708, Republic of Korea; hyohoon.choi@pixel-global.com

**Keywords:** defect inspection, noisy training, printed circuit board, semi-supervised learning

## Abstract

Defect inspection is essential in the semiconductor industry to fabricate printed circuit boards (PCBs) with minimum defect rates. However, conventional inspection systems are labor-intensive and time-consuming. In this study, a semi-supervised learning (SSL)-based model called PCB_SS was developed. It was trained using labeled and unlabeled images under two different augmentations. Training and test PCB images were acquired using automatic final vision inspection systems. The PCB_SS model outperformed a completely supervised model trained using only labeled images (PCB_FS). The performance of the PCB_SS model was more robust than that of the PCB_FS model when the number of labeled data is limited or comprises incorrectly labeled data. In an error-resilience test, the proposed PCB_SS model maintained stable accuracy (error increment of less than 0.5%, compared with 4% for PCB_FS) for noisy training data (with as much as 9.0% of the data labeled incorrectly). The proposed model also showed superior performance when comparing machine-learning and deep-learning classifiers. The unlabeled data utilized in the PCB_SS model helped with the generalization of the deep-learning model and improved its performance for PCB defect detection. Thus, the proposed method alleviates the burden of the manual labeling process and provides a rapid and accurate automatic classifier for PCB inspections.

## 1. Introduction

During the fabrication process of printed circuit boards (PCBs), the presence of fine particles or flaws in copper patterns can cause PCBs to malfunction [1,2]. Therefore, defect screening is a crucial step in the semiconductor manufacturing process for PCBs. Defects can be caused by many factors, including materials, techniques, equipment, and processing substrates [1]. Various inspection systems, such as automated visual inspection (AVI) [2,3], X-ray imaging [4], ultrasonic imaging [5], and thermal imaging [6], have been used to detect defects. Advanced AVI systems utilize high-resolution cameras and defect detection algorithms to inspect PCB surfaces at the panel level [2]. Similar to AVI systems, automatic final vision inspection (AFVI) systems can be used to inspect the surfaces of PCBs at the strip level to detect various types of defects such as cracks, nicks, protrusions, and foreign materials.

Various defect detection algorithms have been utilized in AVI and AFVI systems, such as reference-based [7], rule-based [8,9], and learning-based approaches [10,11]. The majority of these algorithms attempt to find similarities between the reference and inspected images. Nadaf and Kolkure used a subtraction algorithm to detect differences from reference images of typical defect regions on PCBs [7]. The primary purpose of reference-based approaches is to determine precise alignments between the reference and test images [12]. Oguz et al. introduced a design-rule-based defect detection algorithm for verifying the requirements of the geometric design rules of conductor spacings, trace widths, and land widths for digital binary PCB images [9]. Benedek proposed a rule-based framework for generating parent–child relationships based on an intensity histogram to detect soldering defects in PCBs [8].

Recently, the use of learning-based algorithms for defect inspection has increased rapidly. Zhang et al. utilized a support vector machine (SVM) to recognize and classify defect regions by extracting histograms and geometric features from PCB images [11]. Deep-learning approaches have also been employed to assist in filtering false defect images prior to manual verification [2]. Deng et al. proposed an automatic real-time defect classification approach based on deep learning to determine real defects in regions of interest in PCBs [10]. These methods can reduce the burden of manual verification by facilitating the classification of defects. Although deep learning-based defect detection models are expected to alleviate the cost of manual labeling once developed, they require a manual labeling process to supply a significant amount of data for training the model.

Previous studies have employed a transfer-learning approach to reduce the amount of training data [13,14]. Miao et al. employed a cost-sensitive Siamese network based on transfer learning to differentiate defects in PCBs [14]. Imoto et al. developed an automatic defect classification system that employed unreliably labeled data to train a convolutional neural network model to classify multiple defect types [10]. Although previous studies have proven the feasibility of the deep-learning approach for defect detection, the model performance remains limited by the shortage and incorrect labeling of training data. He et al. proposed a semi-supervised learning (SSL)-based model to detect defect locations in a DeepPCB dataset [15]. This approach required pairs of defect-free templates and defective test images for comparison. Shi et al. utilized an adversarial SSL method to train only normal PCB samples and detected extremely small, unknown defects during the test [16]. Although the network can be trained with lesser data for the SSL model, the use of golden template images (i.e., normal images) for training has disadvantages, such as a lack of flexibility for variations (e.g., misalignment and discoloration) and the need to acquire and store numerous reference patterns [1,12].

When defect cases are relatively broad and the differences between defect and non-defect cases are small, the chances of false detection by the defect detection algorithms (e.g., golden template images or machine-learning approaches) of the AFVI system remain high. Hence, following the inspection of the AFVI system, human operators verify whether the defects detected by the AFVI system are true or false to reduce the false detection rate. This manual operation incurs high labor costs and is time-consuming. Manual inspection remains a problem owing to the shortage of labor and expertise. In this study, an SSL approach, PCB_SS, is developed. It can detect defects in PCBs from strip-level images acquired using an AFVI system. The PCB_SS model utilizes labeled data and leverages unlabeled data for training with various augmentations. While varying the amount of labeled data, the performance of the PCB SS model was compared to that of a fully supervised approach, PCB FS, which uses only labeled data. Moreover, the accuracies of the PCB_SS and PCB_FS models were evaluated using noisy (incorrectly labeled) training data in an error-resilience test. To compare the performances of the models for defect detection, extreme gradient boosting (XGBoost) and EfficientNet-B5 were chosen for the machine-learning and deep-learning approaches, respectively.

The highlights of this study are as follows:

(1) An SSL approach is developed for significantly varying defect cases with small differences from the non-defect cases, where the golden template and machine-learning approaches are less effective.
(2) By employing the FixMatch concept with varying augmentations, the PCB_SS model was trained using images classified as defects from the AFVI system with unlabeled and manually labeled target data.
(3) An error-resilience test was performed for false detection. The PCB_SS model outperformed PCB_FS for incorrectly labeled data.
(4) By alleviating the false defect detection from the AFVI systems, PCB_SS can reduce the burden of manual defect detection, requiring intensive labor with expertise in PCB inspection.

## 2. Materials and Methods

### 2.1. Data Generation

#### 2.1.1. Data Acquisition

**Figure 1:**
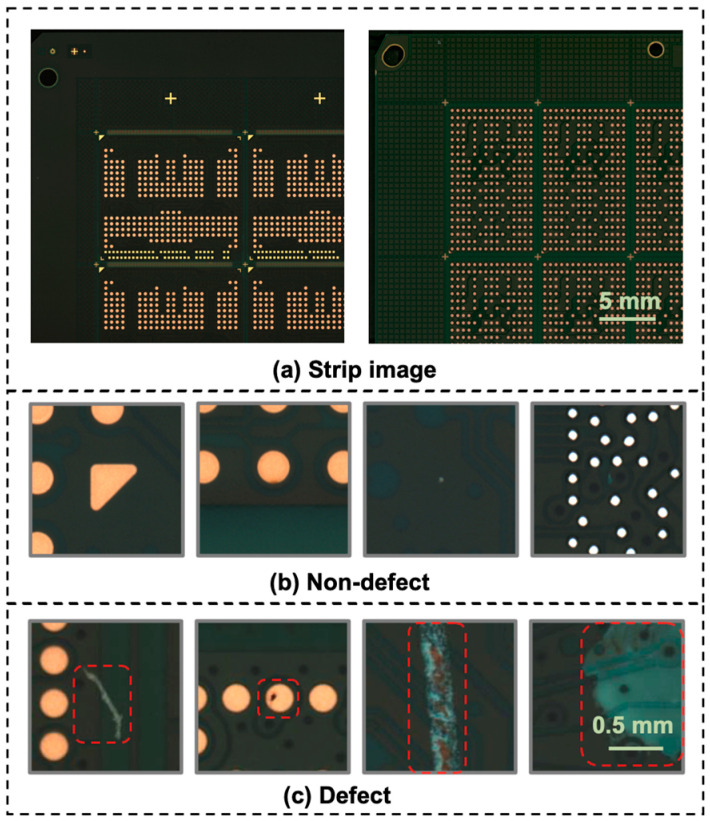
Examples of (**a**) strip-level PCB images and (**b**) non-defect and (**c**) defect images acquired by the AFVI system: the red dashed boxes in (**c**) represent the defect locations on the circuit board.

PCB images were acquired using an AFVI system (S-400SM; Pixel, Inc., Pyeongtaek, Republic of Korea). The field of view of the AFVI is 27.8 × 27.8 mm, and the resolution of the acquired image is 5.4 μm/pixel. The AFVI system automatically detects defect candidates using rule-based methods from the originally captured region for inspection (5120 × 5120 pixels), as shown in Figure 1a. The suspected defects were then saved as smaller-cropped images (250 × 250 pixels), with defects located at the center of the images. These defect-candidate images were further reviewed by experienced human operators using a software tool called an “image-based verification system” and classified as either true or false defects.

Figure 1a shows examples of strip-level PCB images. Figure 1b,c show the data collected as defects from the strip-level PCB images using the AFVI system. Figure 1b shows that the board patch images passed manual screening without defects or with very small imperfections, which did not affect the functional integrity of the board. Figure 1c shows example images with defects of various shapes, sizes, and colors. In this study, 500 different strip-level PCBs were utilized. PCBs had defect issues, with approximately 90% being foreign materials and scratches, while 10% were cracks, nicks, discoloration, and protrusions. Although the defect rates varied for the PCB boards, approximately 40 defect images were acquired per strip-level PCB from the AFVI system, with an average 90% chance of false detection.

#### 2.1.2. Data Preparation

Images of the board patches were resized to 100 × 100 pixels for training and testing. The training data were split into labeled and unlabeled data. For the experiments, the number of labeled data was varied as 250, 500, 1000, 2000, and 4000 (half of the labeled data were from either the defect or non-defect classes). The remaining training data were unlabeled (16,909 images in total) to facilitate the generalization of the PCB_SS model [17]. Validation and test data (500 and 1500 images, respectively) were used to verify and evaluate the model performance during and after training. Table 1 summarizes the data used in this study. To evaluate the error resistances of the models with noisy training data, approximately 0%, 3%, 9%, and 12.5% of the labeled data from the 4000 labeled sets (Label_4000 in Table 1) were relabeled incorrectly, as shown in Table 2.

**Figure 2:**
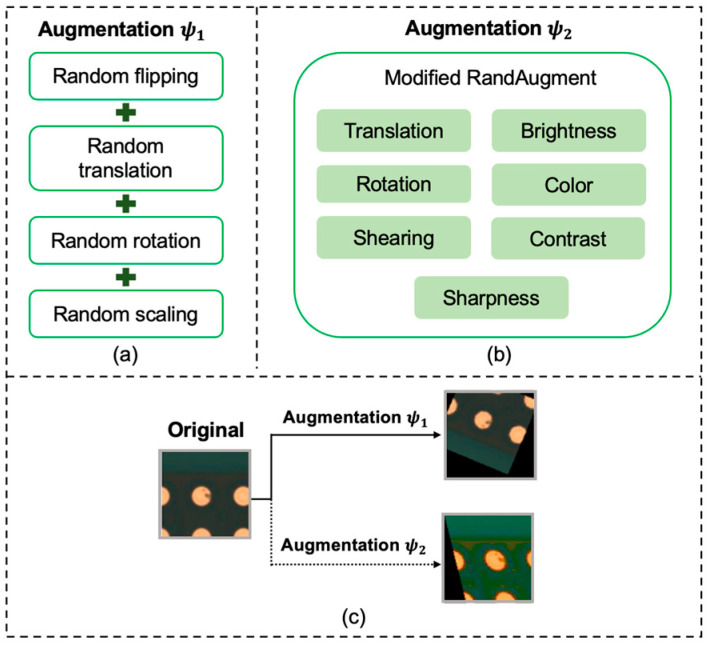
Augmentation process used in this study: (**a**) augmentation for labeled data and unlabeled data for pseudo prediction ($\psi_{1}$), (**b**) augmentation for unlabeled data ($\psi_{2}$), and (**c**) examples of images augmented by the two types.

Inspired by FixMatch [17,18,19], different augmentation strategies have been applied to labeled and unlabeled data. The augmentation methods are illustrated in Figure 2. Figure 2a illustrates the augmentation ($\psi_{1}$) of the labeled data of PCB_SS. Augmentation $\psi_{1}$ comprised random horizontal/vertical flipping, translation, rotation, and scaling. For augmentation $\psi_{2}$, as shown in Figure 2b, two transformations (translation, rotation, shearing, sharpness, contrast, color, and brightness) were randomly selected, along with the magnitudes of these transformations for a batch of images. The same predictions were expected from the unlabeled data when perturbed by two different augmentations (i.e., $\psi_{1}$ and $\psi_{2}$). For the PCB_FS model, augmentation $\psi_{2}$ was used. The range of the transformations is listed in Table 3. For each epoch, all the input training data for the PCB_SS and PCB_FS models were replaced by the augmented data.

### 2.2. Deep-Learning Approach

#### 2.2.1. PCB_FS Model

**Figure 3:**
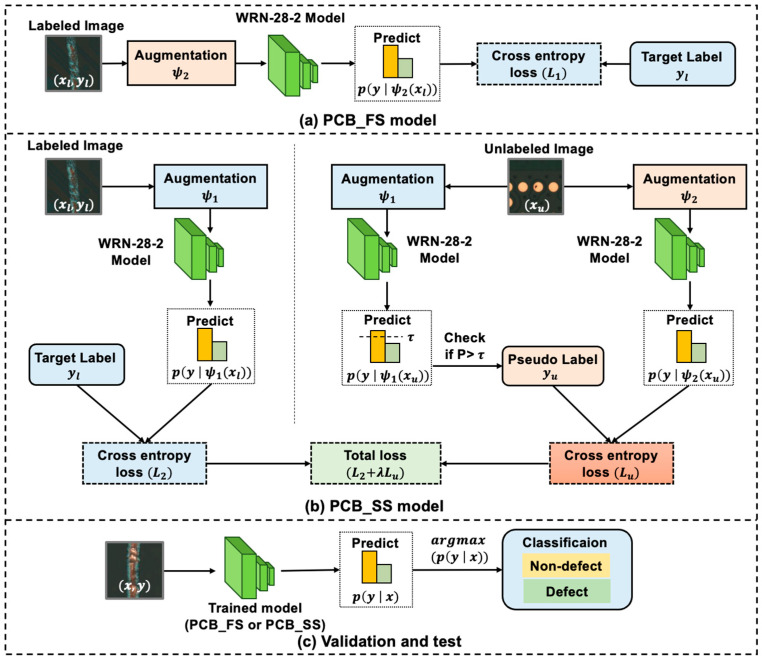
Pipelines for the (**a**) PCB_FS and (**b**) PCB_SS models; (**c**) classification process of the validation and test datasets for the trained model from either (**a**) PCB_FS or (**b**) PCB_SS ($x_{l}$: labeled image, $y_{l}$: target label, $x_{u}$: unlabeled image, $y_{u}$: pseudo-label, *p*: probabilities, τ: threshold of pseudo-label, WRN-28-2: WideResnet-28-2, x, y: validation and test images and their labels).

**Figure 4:**
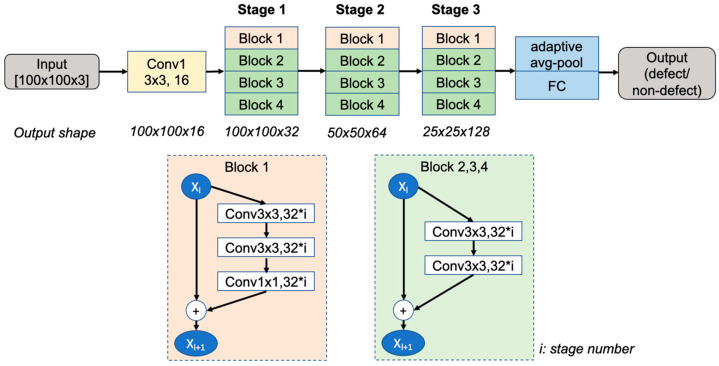
Architecture of WideResNet-28-2 (WRN-28-2) (FC: fully connected layer).

Figure 3a shows the process of training the PCB_FS model using only the labeled data for the baseline. The input image is first augmented ($\psi_{2}$) and fed into the model. WideResNet-28-2 (WRN-28-2) [20] was selected as the network architecture for this study. The architecture of the WRN-28-2 is shown in Figure 4. This model is a wider version of the residual network (ResNet) and consists of 1.47 million parameters, 28 convolution layers, and a widening factor of 2 compared with the original ResNet. The final layer outputs the probabilities of the class labels (i.e., two classes for classifying defects and non-defects) and predicts the class with the highest probability. The loss between the target and predicted classes was defined based on the cross-entropy (CE) loss.

(1) $$L_{1}=\frac{1}{B}\sum_{b=1}^{B}CE\left(y_{l},\;p(y\;|\psi_{2}\left(x_{l}\right)\right))$$

where *B* is the number of labeled images in a batch, $y_{l}$ denotes the target class, and $p(y\;|\psi_{2}\left(x_{l}\right))$ is the probability distribution of each class predicted by the model for the augmented $\psi_{2}$ version of the labeled image ($x_{l}$).

#### 2.2.2. PCB_SS Model

The PCB_SS model used labeled data, as in the PCB_FS model, as shown in Figure 3a, with augmentation $\psi_{1}$ on the labeled data and leveraged the unlabeled data to improve generalization, as shown in Figure 3b. For unlabeled data, after applying augmentation $\psi_{1}$, the model predicted the class of the image with the highest probability and assigned a pseudo-label if the probability was higher than a predefined threshold τ. The pseudo-label was used to compare the predicted output class from the augmentation $\psi_{2}$ to compute the unsupervised loss function. The supervised and unsupervised loss functions were also calculated based on the CE loss. The implemented condition for the class with the highest probability of pseudo-labeling is as follows:

(2) $$L_{2}=\frac{1}{B}\overset{B}{\underset{b=1}{\sum }}CE\left(y_{l},\;p(y\;|\psi_{1}\left(x_{l}\right)\right))$$

(3) $$L_{u}=\frac{1}{\mu B}\overset{\mu B}{\underset{b=1}{\sum }}1(\max\left(q\right)>\tau )CE\left(y_{u},p(y\;|\;\psi_{2}\left(x_{u}\right)\right))$$

(4) $$q=p(y\;|\psi_{1}\left(x_{u\;}\right))$$

(5) $$y_{u}=argmax\left(q\right)$$

where $L_{2}$ and $L_{u}$ denote the supervised and unsupervised loss functions, respectively; $p(y\;|\psi_{1}\left(x_{l}\right))$ is the probability distribution of each class predicted by the model for the augmented $\psi_{1}$ version of the labeled image ($x_{l}$); *μ* is the relative coefficient of the unlabeled images in a batch; $y_{u}$ is the pseudo-label; and q denotes the probability distribution of each class predicted by the model for the augmented $\psi_{1}$ version of the unlabeled image ($x_{u}$). The total loss was calculated as follows:

(6) $$L=L_{2}+\lambda L_{u}$$

where λ is the coefficient of unlabeled loss, which is a fixed scalar hyperparameter that determines the ratio of unsupervised to supervised losses. The value of λ was set to 1 in this study (Table 4).

#### 2.2.3. Network Training

Table 4 lists the hyperparameter configuration used to train the PCB_FS and PCB_SS models. The parameters of the convolutional and fully connected layers were initialized by the He and Xavior (Glorot) initializations, respectively [21,22]. The total loss in a batch was backpropagated and used to update the model weights using stochastic gradient descent (SGD) optimization with Nestorov momentum [23]. To accelerate the training, the learning rate was initially set to 0.001 for both models and scheduled during training using a cosine learning rate decay [18,23]: $\eta \cos\left(\frac{7\pi k}{16K}\right),\;$ where η, k, and K denote the initial learning rate, the current training step, and the total number of training steps, respectively. The threshold (τ) for the pseudo-label of the unlabeled data was 0.9. After updating the gradients of the data in a batch, the parameters of the model were averaged over the training iterations using the exponential moving average (EMA) method to avoid fluctuation by applying a larger coefficient (α = 0.999) to the recent weights [24].

(7) $$\theta_{t}^{\prime }=\alpha \theta_{t-1}^{\prime }+\left(1-\alpha \right)\theta_{t}$$

where α denotes the smoothing coefficient (EMA decay), $\theta_{t}^{\prime }$ and $\theta_{t-1}^{\prime }$ are the weights of the EMA model in the current and previous steps, respectively, and $\theta_{t}$ denotes the gradient updated model at the current step. The validation set was evaluated after a training period of 256 iterations. The weight decay coefficient is a regularization factor used to decrease the weights during the optimization.

The pseudocode of the PCB_SS model is as follows (Algorithm 1).
**Algorithm 1** Pseudocode of PCB_SS model(1)***Input*:** Labeled batch (*L*), unlabeled batch (*U*), confidence threshold (τ), unlabeled data ratio (μ), unlabeled loss weight (λ)(2)Initialize the WRN-28-2 model(3)Copy initial parameters of the model to EMA model(4)**for** epoch = 1 **to** EPOCHs **do**(5)**for** batch = 1 **to** eval_steps **do**(6)    Apply augmentation $\psi_{1}$ to L and U.(7)    Compute the loss of L by Equation (2).(8)    Assign pseudo-label for U by Equations (4) & (5).(9)    Apply augmentation $\psi_{2}$ to U.(10)  Compute the loss of U by Equation (3)(11)  Update model parameter by SGD optimization.(12)  Update EMA model by transferring the updated model’s parameters by Equation (7).(13)**end for**(14)Calculate the loss and accuracy on validation data(15)**end for**(16)**Output**: the trained network parameters with the best accuracy on validation data

### 2.3. Performance Evaluation

To evaluate the performance of the PCB_FS and PCB_SS models, all models were trained three times. The classification process is illustrated in Figure 3c. To compute the number of misclassified data points over the test data, the error rate was computed as follows:

(8) $$error\;rate=\frac{FP+FN}{TP+FP+TN+FN}*100\;\left(\%\right)$$

where FP,FN,TP, and TN denote the number of false-positives, false-negatives, true-positives, and true-negatives, respectively, for the defect class. The average error rates for the test data from the three training sessions were calculated.

The best PCB_FS and PCB_SS models were selected from the top three trained models and compared in terms of recall, precision, area under the curve (AUC) score, confusion matrix, and receiver operating characteristics (ROC) curve [25]. Detecting a defect case has a higher priority than detecting a non-defect case. Thus, the recall (i.e., true-positive rate) of the defect class is an important metric for verifying the validity of the proposed method. Precision indicates the proportion of correct defect predictions for all the defects classified by the model. The ROC curve shows the true-positive rate against the false-positive rate by varying the probability thresholds for the prediction. To visualize the regions in which the model concentrates for the decision, gradient-weighted class activation mapping (Grad-CAM++) [26] was utilized.

To further evaluate the performance of the proposed model, a machine-learning algorithm was applied and compared. First, the features were extracted and quantized using a scale-invariant feature transform (SIFT) algorithm [27]. XGBoost (learning rate 0.15, gamma 0.4, maximum depth 10, and minimum sum of instance weight 5) used the extracted features [28,29]. Grid-search cross-validation was used to set the optimal hyperparameters for the best performance [30]. In addition, the performance of the proposed model was compared with that of a deep-learning classifier. EfficientNet-B5 was utilized with the same hyperparameters as in the PCB_FS model.

## 3. Results

### 3.1. Learning Curves

**Figure 5:**
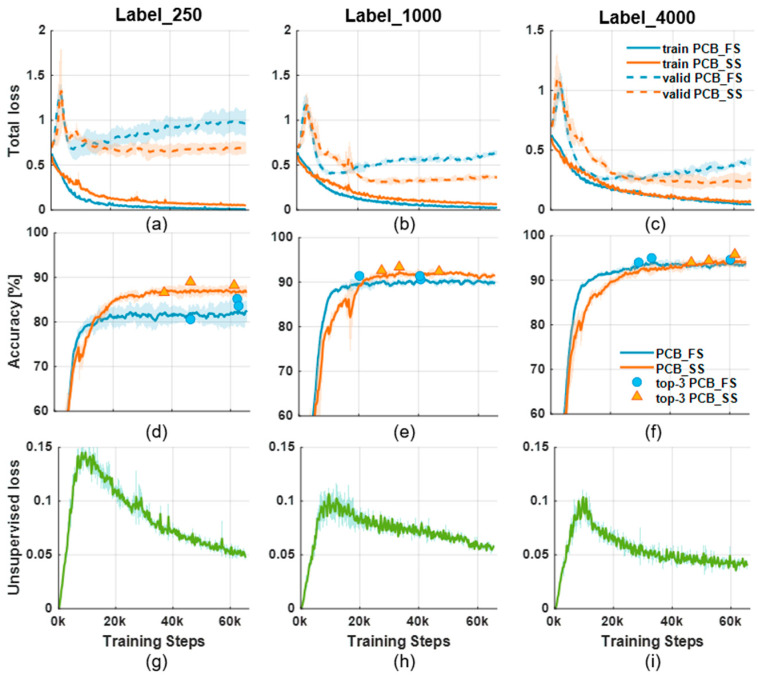
Learning curves during training for Label_250, Label_1000, and Label_4000: (**a**–**c**) total losses on training data (solid lines) and validation losses (dotted lines), (**d**–**f**) validation accuracies (circle and triangle: training steps of top three accuracies for PCB_FS and PCB_SS, respectively), and (**g**–**i**) unsupervised losses on the unlabeled data of the PCB_SS model.

Figure 5 shows the learning curves of the PCB_FS and PCB_SS models when trained with 250, 1000, and 4000 labeled images. Figure 5a–c show the total losses during the training of the PCB_FS and PCB_SS models using 250, 1000, and 4000 labeled data, respectively. Overall, increasing the number of labeled data does not result in significant differences in the training losses for the PCB_FS and PCB_SS models. However, the validation loss decreased noticeably with increasing numbers of labeled data. The validation loss of the PCB_SS model was lower than that of the PCB_FS model because the PCB_SS model was generalized by leveraging unlabeled data. The accuracies shown in Figure 5d–f for the validation set also show that the PCB_SS model outperformed the PCB_FS model. Figure 5g–i show the unsupervised losses from the PCB_SS model during training. In the early training stage (0–10,000 training steps), the unsupervised losses increase because a large number of unlabeled data have a higher probability of a false class than the threshold value (see Equation (3). This indicates that the model parameters were initially learned from the features extracted from the labeled data. Subsequently, the PCB_SS model starts to learn the features from the unlabeled data to minimize the unsupervised loss gradually.

### 3.2. Performance Evaluation

Table 5 shows the error rates of the test data inferred from the PCB_FS and PCB_SS models trained with different numbers of labeled data. Overall, the error rate decreased when the models were trained using a larger number of labeled samples. The PCB_SS model outperformed the PCB_FS model in all the cases. The error rates of the PCB_SS model were lower than those of the PCB_FS model by 8.25% and 3.40% for Label_250 and Label_4000, respectively. In addition, the PCB_SS model with Label_500 (a mean error rate of 11.98%) achieved results comparable to those of the PCB_FS model with Label_4000 (a mean error rate of 11.22%).

**Figure 6:**
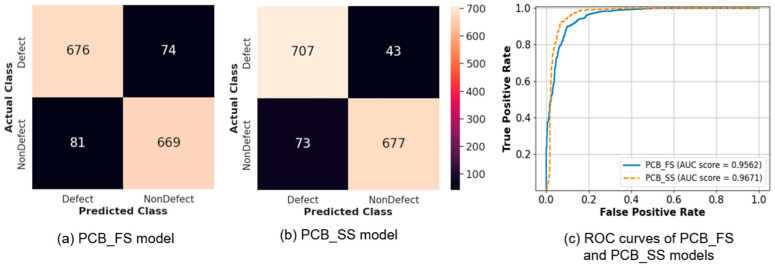
Confusion matrix of the (**a**) PCB_FS and (**b**) PCB_SS models, and (**c**) ROC curves for the two models (solid: PCB_FS, dotted: PCB_SS).

The best models for PCB_FS and PCB_SS learning were obtained from Label_4000. The confusion matrices for the best PCB_FS and PCB_SS models are shown in Figure 6a,b, and the ROC curves for these models are shown in Figure 6c. The number of misclassified images was higher for the PCB_FS model in both classes (defects and non-defects). Table 6 summarizes the performance of the proposed models (PCB_SS and PCB_FS), XGBoost, and EfficientNet-B5. Deep-learning models obtained better classification results than machine-learning algorithms. The proposed PCB_SS model outperformed XGBoost, EfficientNet, and PCB_FS by 15.2, 4.1, and 2.9%, respectively, in terms of accuracy. Overall, the proposed PCB_SS model achieved greater recall, precision, and AUC scores than the other models, as shown in Table 6.

**Figure 7:**
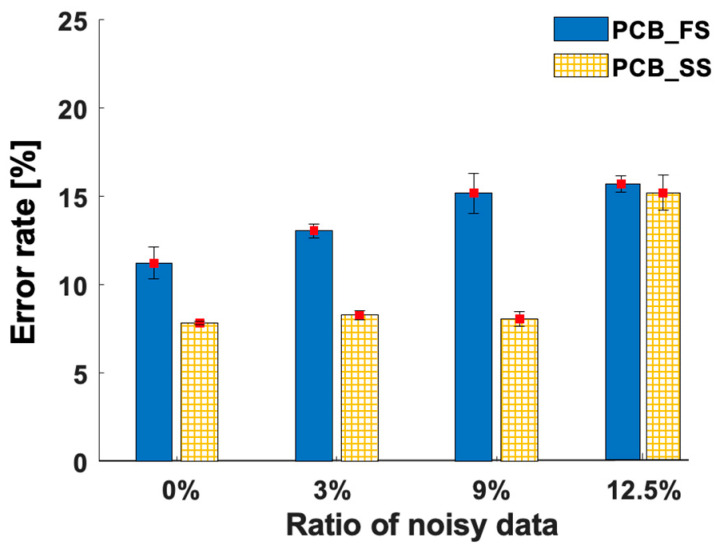
Error rates (%) of the test data with different ratios of noisy data.

Table 7 and Figure 7 illustrate the error rates of the test data from the error-resilience test of the deep-learning models. The error rate of the PCB_FS model increased by 4% when the noisy data ratio was increased to 9%. Meanwhile, the error rate of the PCB_SS model was relatively consistent. The increase in the error rate of the PCB_SS model was less than 0.5%, whereas the noisy data ratio varied from 0 to 9%. When the noisy data ratio increased to 12.5%, the error rate of the PCB_SS model was similar to that of the PCB_FS model. Thus, the PCB_SS model was more robust to noisy data (i.e., incorrectly labeled data) than the PCB_FS model when the noisy data ratio was lower than 12.5%.

In the error-resilience test, unsupervised loss helped the PCB_SS model resist noisy data in the labeled set. Meanwhile, the PCB_FS model performed worse when incorrectly labeled data were present in the training set. Table 8 lists the error rates of the unlabeled data from the error-resilience tests of the models. The error rates of the test data were associated with those of the unlabeled data. When the noisy data ratio is from 0% to 9%, the error rates of the unlabeled and test data of the PCB_SS model are consistent, while those of the PCB_FS model increase corresponding to noisy data ratios of 4.6% and 3.9% for unlabeled data (Table 8) and test data (Table 7), respectively. With a noisy data ratio of 12.5%, the error rate of the unlabeled data increased by 3.3% (Table 8), and that of the test data increased by 7.1% (Table 7). This result also proves the effect of incorrectly labeled data on the pseudo-labels assigned by the model.

### 3.3. Parameter Optimization

Augmentation is a critical factor in the model performance. Table 9 lists the error rates of the PCB_FS and PCB_SS models with various augmentations in the Label_4000 dataset. For PCB_SS, the augmentation $\psi_{1}$ on the labeled and unlabeled data for pseudo-label prediction was replaced by no augmentation (∅) and augmentation $\psi_{2}$. With no augmentation, the performance of both models degraded. The PCB_FS and PCB_SS models yielded the best performance with augmentation $\psi_{2}$ and augmentation $\psi_{1}$, respectively.

Table 10 lists the error rates along with the recall and precision of the defect class of the PCB_SS model with varying thresholds (0.4, 0.7, 0.9, and 1.0). Assigning an appropriate threshold value (τ = 0.9, Table 10) allows high-quality unlabeled images, with which the model can make inferences with high confidence, to contribute to the reduction in unsupervised loss. A high threshold is expected to improve the performance of the pseudo-labeling [18]. Considering the abrupt error rate increase at 1.0 and the highest recall value at 0.9, a threshold of 0.9 was chosen in this study.

The coefficient of unlabeled loss (λ) is another parameter that affects the performance of the model. Initially, the loss of labeled data was more critical than that of unlabeled data. However, the effect of unlabeled data increases during training [18]. In the early epochs, most of the pseudo-labels of the unlabeled data were incorrect (i.e., $\max\left(q\right)<\tau$ in Equation (3)). As the model learns more features from labeled data, the probability of a pseudo-label increases due to the neighbor distribution, and more unlabeled data can exceed the threshold (i.e., $\max\left(q\right)>\tau$ in Equation (3)). Therefore, the effect of the unlabeled loss increases during training, regardless of the initial value of λ.

### 3.4. Gradient Visualization (Grad-CAM)

**Figure 8:**
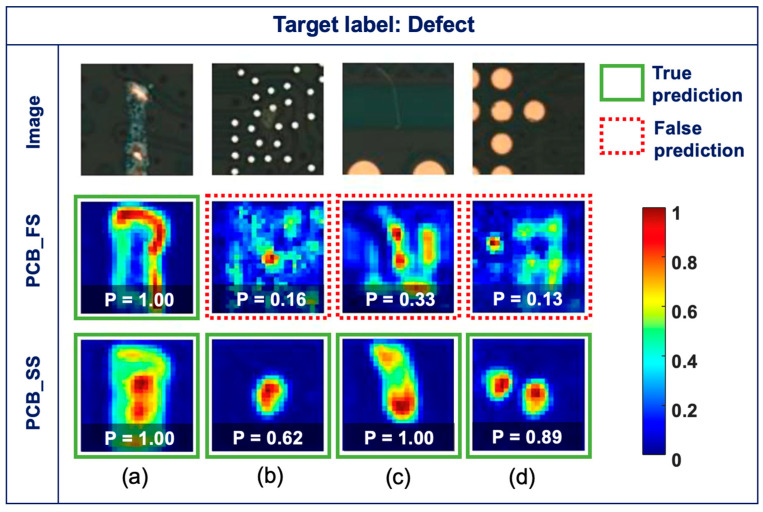
Original images (top row) and Grad-CAM maps obtained using the PCB_FS model (middle row) and PCB_SS model (bottom row) (**a**) true predictions for both PCB_FS and PCB_SS models, (**b**–**d**) false and true predictions for PCB_FS and PCB_SS models, respectively (green solid box: true prediction, red dotted box: false prediction).

Figure 8 shows the original images (top row) and Grad-CAM maps obtained from the PCB_FS (middle row) and PCB_SS (bottom row) models for the defect classes and their corresponding probabilities. Figure 8a shows the images classified correctly by the PCB_FS and PCB_SS models with high confidence (i.e., the probabilities of the PCB_FS and PCB_SS models are 1.00). While the Grad-CAM maps from the PCB_FS model focused on the defect boundaries, those from the PCB_SS model focused on the defects more precisely. Figure 8b–d show hard cases of defects (e.g., unclear objects, thin scratches, and small pin holes). The PCB_FS model provides false predictions (i.e., classified as non-defects), which correspond to an incorrect focus from Grad-CAM, and prediction probabilities of 0.16, 0.33, and 0.13 for the input images in Figure 8b–d, respectively. In contrast, the PCB_SS model showed high activation at the defect locations with true predictions and prediction probabilities of 0.62, 1.00, and 0.89 for the input images in Figure 8b–d, respectively. Although the models were trained without information on the defect locations, decisions from the models were made based on the detected features.

## 4. Discussion

This study demonstrated the robustness of the PCB_SS model for defect classification when the labeled data were limited or incorrectly labeled for training. The proposed PCB_SS model can benefit from the inspection procedure by reducing the burden of manual inspection. Although the SSL approach for the PCB_SS model was adopted from the FixMatch model [18], the applications of which are limited to object-specific classification, the defect detection in this study was related to feature-related recognition rather than object detection. Furthermore, optimal augmentations for labeled and unlabeled data were selected to improve the effectiveness of the model in increasing the diversity of data without label changes (Table 9). In the SSL approach, it is difficult for the model to generate a neighbor connection between the perturbed versions of the unlabeled data and labeled data with no augmentation (∅) or augmentation $\psi_{2}$. Thus, the labeled and unlabeled data for the pseudo-label prediction of the PCB_SS model were augmented by augmentation $\psi_{1}$. Augmentation $\psi_{1}$, as shown in Figure 2a, can increase the variation in the dataset for various types, shapes, and sizes of defects. The training process for the labeled data can be accelerated because of the model with different augmented data after each evaluation step [19]. Labeled data are critical for creating the underlying feature space, and SSL takes advantage of prior knowledge of the domain and data distribution to relate the data and labels to improve the classification performance [17,18,24,31,32]. For the PCB_FS model, augmentation $\psi_{2}$, as shown in Figure 2b, comprehensively generates diverse variations in the training dataset and reduces the search space of the transformations [19,32]. The computational time for inspecting a strip-level PCB image was 164 GFLOPs, which required 25 ms, using the resources utilized in this study.

Table 6 shows a comparison of the proposed model with the machine-learning (XGBoost) and deep-learning (EfficientNet) approaches. The results show that the proposed model is efficient for PCB defect detection, especially when the differences between the defect and non-defect images are small and the defect cases vary widely. Because the percentage of misclassified defect patches from the AFVI system is high (90%, on average), it is crucial to utilize a deep-learning approach to enhance the inspection process to alleviate the false detection of the AFVI system and thus further require manual validation. Based on this comparison, the current backbone model (i.e., WRN-28-2) is superior to EfficientNet. However, various deep-learning models can be selected as the backbone for the future work.

Training the network using unlabeled data and adding unsupervised loss made it possible to generalize the parameters of the PCB_SS model (i.e., overfitting was avoided) (Figure 5). This generalization of the model can help classify unseen data, which were not previously introduced into the training data, based on the information learned from the unlabeled data. As shown in Figure 5, the difference in the validation losses between the PCB_FS and PCB_SS models was higher than the difference in the validation accuracies between the models. This can occur when the model is less certain about the prediction, resulting in a higher loss even though the model predicts correctly. The Grad-CAM maps (Figure 8) visually confirmed that the model extracted features from the defect regions for decision making. Furthermore, the augmentation process did not affect the decision features, although it modified the training images. In addition, the PCB_SS model can resist noisy data better than the PCB_FS model (Table 7 and Figure 7).

**Figure 9:**
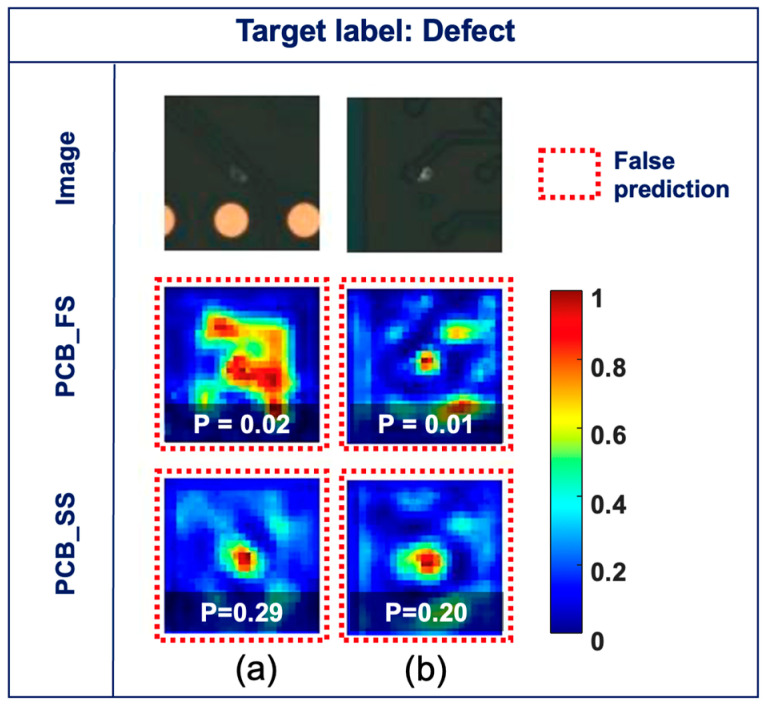
Examples of defect images (**a**,**b**) misclassified by both models.

In this study, the error rate of the unlabeled data with a probability exceeding the predefined threshold (τ) for the PCB_SS model decreased as the number of labeled data increased from 250 to 4000 (15.94% and 3.95% for Label_250 and Label_4000, respectively). This proves that a larger number of correctly labeled data can provide more guidance for the learning procedure, which also improves the clustering process for unlabeled data. The quality of the pseudo-labels predicted from the unlabeled data is critical for obtaining a high accuracy with the PCB_SS model. Further investigations can be conducted on the dynamic threshold to adaptively select appropriate unlabeled samples to avoid degradation of the overall performance. As wider variations were expected for defect cases than for non-defect cases, the proportions of unlabeled data for defects and non-defects were imbalanced (i.e., more defects than non-defect data), as shown in Table 1. The results of the confusion matrices (Figure 6) demonstrated that the reduction in the false prediction of the defect class (42%) between the PCB_FS and PCB_SS models was more significant than that of the non-defect class (10%). Considering that the main objective of inspection is not to miss the defects in PCBs, imbalanced data can improve the classification performance. As demonstrated in the confusion matrix (Figure 6), the PCB_SS model had fewer false predictions than the PCB_FS model for the test set. Figure 9 shows the Grad-CAM maps of the two defect images that were misclassified by the PCB_FS and PCB_SS models. Both defects are related to a foreign object (e.g., dust), which is difficult to detect visually. Although the images were misclassified, the Grad-CAM maps showed that the PCB_SS model still concentrated on the defect location. However, the prediction results were also affected by the low activation (approximately 0.3–0.4) of the surrounding regions. Further analysis must be conducted to develop a model that is less affected by the activation of the surrounding region [33].

The current study had several limitations. The PCB_SS model expects the ROI images to locate defect candidates from the AFVI system. Thus, it is necessary to acquire patch images to apply the current approach to whole PCBs or assembled PCBs (i.e., PCBAs). When new circuit patterns are tested, the proposed model may require further updates by training with additional circuit-pattern images. The new training process can be performed efficiently by utilizing transfer learning from the previous weights of the model. For the best Label_4000 model, the percentage of unlabeled data with a probability higher than the threshold (τ) was only 88%. The PCB_SS model requires further improvement by creating close connections between the labeled and unlabeled data [34].

The results of this study demonstrate that the PCB_SS model performs effectively when a limited number of data are labeled or a portion of them are incorrectly labeled. The proposed model was most effective when the defect cases varied significantly with small changes from the non-defect cases. Combining this method with the AFVI system is expected to significantly minimize the need for manual inspection for false detections and potentially reduce the cost incurred in the PCB manufacturing process.

## 5. Conclusions

The proposed PCB_SS model can effectively detect defects in PCB images when trained using labeled and unlabeled data. The unsupervised loss of unlabeled data perturbed by two different augmentations contributes to improving the performance of the PCB_SS model for cases with data-labeling shortages or errors. Further research should focus on investigating and building a robust SSL model for inspection systems to analyze multiple types of PCB defects and error resistance with higher proportions of noisy data. In addition, wider and deeper variants of WRN-28-2 and advanced models, such as Transformers, will be employed as the backbone to improve the capacity of the deep-learning model [35,36,37]. Furthermore, this study can be extended to classify various types of PCB [13,38].

## Figures and Tables

**Table 1:** Dataset used in this study.

Purpose	Set	Defect	Non-Defect	Total
Train	Label_250	125	125	250
	Label_500	250	250	500
	Label_1000	500	500	1000
	Label_2000	1000	1000	2000
	Label_4000	2000	2000	4000
	Unlabeled	10,791	6118	16,909
Validation		250	250	500
Test		750	750	1500
Total		13,791	9118	22,909

**Table 2:** Dataset for the model error-resilience test.

Set with Noisy	Defect		Non-Defect		Total
	Correct	Noisy	Correct	Noisy	
0%	2000	0	2000	0	4000
3%	1940	60	1940	60	4000
9%	1820	180	1820	180	4000
12.5%	1750	250	1750	250	4000
Unlabeled	10,791		6118		16,909

**Table 3:** List of transformations and their ranges.

Transformation	Range
Rotation $(\psi_{1}\;\&\;\psi_{2}$)	[−30, 30]
Translation $(\psi_{1}$)	[−0.3, 0.3]
Translation $(\psi_{2}$)	[−0.45, 0.45]
Scaling	[0.9, 1.1]
Shearing	[−0.3, 0.3]
Brightness Color Contrast Sharpness	[0.1, 1.9]

**Table 4:** Hyperparameter configuration for the training process.

Configuration	PCB_FS Model	PCB_SS Model
Loss function	Cross entropy (Equation (1))	Cross entropy-based (Equation (6))
Optimizer	SGD	SGD
Initial learning rate $\left(\eta \right)$	0.001	0.001
Learning rate schedule	$\eta \cos\left(\frac{7\pi k}{16K}\right)$	$\eta \cos\left(\frac{7\pi k}{16K}\right)$
Batch size	16	16 (labeled) & 144 (unlabeled)
Total number of training steps	65,536	65,536
Number of evaluation steps	256	256
Threshold (τ)	-	0.9
Coefficient of unlabeled batch size (μ)	-	9
Coefficient of unlabeled loss (λ)	-	1
EMA decay coefficient (α)	0.999	0.999
Weight decay coefficient	0.0005	0.0005

**Table 5:** Error rates (%) of test data with different numbers of labeled data (Best result in bold).

Model	Label_250	Label_500	Label_1000	Label_2000	Label_4000
PCB_FS	22.27 ± 0.33	18.82 ± 0.31	16.36 ± 0.49	13.00 ± 0.55	11.22 ± 0.91
PCB_SS	**14.02 ± 0.11**	**11.98 ± 0.88**	**12.29 ± 0.60**	**8.71 ± 0.44**	**7.82 ± 0.08**

**Table 6:** Classification performances of the PCB_FS and PCB_SS models and the machine-learning (XG-Boost) and deep-learning (EfficientNet) approaches (Best result in bold).

Metric	XG-Boost	EfficientNet	PCB_FS	PCB_SS
Accuracy (%)	80.07	88.60	89.67	**92.27**
Recall	0.80	0.88	0.90	**0.94**
Precision	0.81	0.88	0.89	**0.91**
AUC	0.96	0.95	0.96	**0.97**

**Table 7:** Error rates (%) of test data with different ratios of noisy data (Best result in bold).

Model	Noisy (0%)	Noisy (3%)	Noisy (9%)	Noisy (12.5%)
PCB_FS	11.22 ± 0.91	13.04 ± 0.38	15.18 ± 1.15	15.60 ± 0.48
PCB_SS	**7.82 ± 0.08**	**8.27 ± 0.25**	**8.04 ± 0.42**	**15.11 ± 0.99**

**Table 8:** Error rates (%) of unlabeled data with different noisy data ratios (Best result in bold).

Model	Noisy (0%)	Noisy (3%)	Noisy (9%)	Noisy (12.5%)
PCB_FS	11.29 ± 1.34	13.2 ± 0.27	15.88 ± 1.01	16.85 ± 0.27
PCB_SS	**8.37 ± 0.49**	**8.18 ± 0.56**	**8.96 ± 0.84**	**14.22 ± 1.31**

**Table 9:** Error rates of test data of PCB_FS and PCB_SS models with varying augmentations (∅: no augmentation, and $\psi_{1}$ and $\psi_{2}$ are selected augmentations, Best result in bold).

Augmentation	PCB_FS	PCB_SS
∅	13.6 ± 0.23	15.78 ± 0.867
$\psi_{1}$	14.98 ± 1.01	**7.82 ± 0.08**
$\psi_{2}$	**11.2 ± 0.91**	9.36 ± 1.47

**Table 10:** Test set metrics of the PCB_SS model using different thresholds (τ) (Best result in bold).

Threshold (τ)	Recall	Precision	Error Rate
0.4	0.91 ± 0.02	0.94 ± 0.01	7.44 ± 0.64
0.7	0.91 ± 0.02	0.94 ± 0.01	6.95 ± 0.65
0.9	**0.93 ± 0.01**	0.92 ± 0.01	7.82 ± 0.08
1.0	0.85 ± 0.02	0.84 ± 0.01	**15.36 ± 0.51**

## Data Availability

The data presented in this study are available upon request from the corresponding author.
